# Unsafe abortion in Kenya: a cross-sectional study of abortion complication severity and associated factors

**DOI:** 10.1186/s12884-015-0459-6

**Published:** 2015-02-15

**Authors:** Abdhalah Kasiira Ziraba, Chimaraoke Izugbara, Brooke A Levandowski, Hailemichael Gebreselassie, Michael Mutua, Shukri F Mohamed, Caroline Egesa, Elizabeth W Kimani-Murage

**Affiliations:** African Population and Health Research Center (APHRC), APHRC Campus, Manga Close, off Kirawa road, Kitisuru, P.O. Box 10787–00100, Nairobi, Kenya; Ipas, P.O. Box 9990, Chapel Hill, NC 27515 USA

**Keywords:** Unsafe abortion, Kenya, Maternal mortality, Complication, Severity, Termination of pregnancy

## Abstract

**Background:**

Complications due to unsafe abortion cause high maternal morbidity and mortality, especially in developing countries. This study describes post-abortion complication severity and associated factors in Kenya.

**Methods:**

A nationally representative sample of 326 health facilities was included in the survey. All regional and national referral hospitals and a random sample of lower level facilities were selected. Data were collected from 2,625 women presenting with abortion complications. A complication severity indicator was developed as the main outcome variable for this paper and described by women’s socio-demographic characteristics and other variables. Ordered logistic regression models were used for multivariable analyses.

**Results:**

Over three quarters of abortions clients presented with moderate or severe complications. About 65 % of abortion complications were managed by manual or electronic vacuum aspiration, 8% by dilation and curettage, 8% misoprostol and 19% by forceps and fingers. The odds of having moderate or severe complications for mistimed pregnancies were 43% higher than for wanted pregnancies (OR, 1.43; CI 1.01-2.03). For those who never wanted any more children the odds for having a severe complication was 2 times (CI 1.36-3.01) higher compared to those who wanted the pregnancy then. Women who reported inducing the abortion had 2.4 times higher odds of having a severe complication compared to those who reported that it was spontaneous (OR, 2.39; CI 1.72-3.34). Women who had a delay of more than 6 hours to get to a health facility had at least 2 times higher odds of having a moderate/severe complication compared to those who sought care within 6 hours from onset of complications. A delay of 7–48 hours was associated with OR, 2.12 (CI 1.42-3.17); a delay of 3–7 days OR, 2.01 (CI 1.34-2.99) and a delay of more than 7 days, OR 2.35 (CI 1.45-3.79).

**Conclusions:**

Moderate and severe post-abortion complications are common in Kenya and a sizeable proportion of these are not properly managed. Factors such as delay in seeking care, interference with pregnancy, and unwanted pregnancies are important determinants of complication severity and fortunately these are amenable to targeted interventions.

## Background

Globally, unsafe termination of pregnancy (TOP) remains a major public health problem and World Health Organization (WHO) reports that 21.6 million unsafe abortions occurred in 2008 [1]. Annually, an estimated 8.5 million women suffer from complications of unsafe TOP, resulting in 47,000 maternal deaths [1,2]. The majority of unsafe pregnancy terminations occur in the developing countries with the most severe morbidity and mortality occurring in sub-Saharan Africa [1,3]. In a recent national survey, it was estimated that 465,000 induced abortions occurred in Kenya in 2012 (48 per 1000 women of reproductive age) [4]. Because of the restrictive anti-abortion laws and limited access to quality health care, most induced abortions in sub-Saharan Africa, including Kenya, are unsafe and contribute to the high morbidity and mortality burden [3,5,6]. Under the new Kenyan constitution, induced abortion can be provided for certain indications as determined by a trained health care provider. However, this law is yet to be fully operationalized and most providers have not been trained to provide safe TOP. This means that many of the induced abortions taking place now are unsafe. Additionally, while most induced abortions follow unintended pregnancies, the unmet need for family planning remains high in many countries including Kenya [7].

In Kenya, the maternal mortality ratio currently stands at 488 deaths (95% CI 333–643) per 100,000 live births and many of these are due to abortion complications [8]. A study conducted in Kenyatta National Hospital showed a maternal mortality ratio of 921 deaths per 100,000 live births and over a third (36%) of these occurred following an unsafe TOP [9]. From 2003 to 2008, a maternal death ratio of 740 deaths (95% CI 651–838) of women of childbearing age per 100,000 live births was estimated for Western Kenya with 78% of the deaths being attributable to unsafe induced abortions [10]. Another study in two slums of Nairobi informal settlements estimated a maternal mortality ratio of 706 per 100,000 live births and a third of these were due to abortion complications [11].

While abortion complications are recognized as a major contributor to maternal deaths, there are no recent national estimates of the burden of abortion and its presentation in Kenya. The last major study that estimated the incidence of abortion was conducted in 2003 and only covered higher level public facilities [12]. In the context of high incidence of induced abortion in Kenya, this study set out to generate evidence on the severity of post-abortion complications. The severity of an abortion complication gives an indication of the risk of death or near-miss and this is important for targeted interventions. The study examined both induced abortions and spontaneous miscarriages, and categorized according to the responses and clinical findings. Based on the methodology used and stigma associated with reporting an induced abortion, it was not possible to determine if induced abortions were safe or not. Specifically, this paper characterizes the severity of abortion complications and factors associated with it to inform policy, planning and implementation of interventions aimed at preventing and managing abortion complications.

## Methods

Data used in this paper came from a 30-day prospective abortion-related morbidity survey conducted in a nationally-representative sample of health care facilities in Kenya. The sample of participating facilities was selected from a master facility list of all health care facilities in Kenya and stratified on the basis of level of service and geo-political region. A health care facility level is a designation of functionality as defined by the Ministry of Health in Kenya and outlined in the Kenya Essential Package for Health [13]. Level I is the lowest, at the community level, while Level VI is the highest level of health care provision in the country. For this study, all Levels V-VI facilities were included and a random sample of facilities was selected from Levels II-IV facilities, with sampling fractions ranging from 0.18-0.36 in Level IV; 0.08-0.15 in Level III; and 0.05-0.19 in Level II.

A total of 350 facilities were sampled, of which 326 participated (93%); and out of these 292 health care facilities provided data used in the study. All women presenting at a participating facility for TOP or treatment for abortion complications were interviewed using a pretested tool. Before any interviews were conducted, informed verbal consent was sought from each client. Data collected included socio-demographic characteristics, presenting complaints, history of interference with the pregnancy, last normal menstruation period, symptoms related to complications, diagnoses, procedures performed and final clinical outcome. Over the 30 days period of the survey, a total of 3,153 women reported to the participating health facilities seeking abortion-related care. Out of these 2,625 (86.4%- weighted) were seeking post-abortion care (PAC) while the rest (528) were seeking termination of pregnancy (TOP). This analysis is restricted to women seeking treatment for complications of either a spontaneous miscarriage or unsafe TOP and with a gestational age less than 24 weeks (n = 2,625). Gestational age was estimated using the last menstrual period date. For those missing this information, the provider’s estimation of gestation age was used. Based on criteria for measuring severity previously used elsewhere [12,14,15], a set of complications were assessed and used to generate a level of severity indicator for each woman treated for PAC (Table 1). The severity variable has three categories: low/mild complication, moderate complication and severe complication. The total number of deaths in 30 days (7 deaths) was multiplied by 12.2 to obtain a yearly estimate of deaths due to unsafe abortion. A case-fatality rate per 100,000 unsafe abortions was calculated by dividing the number of total deaths in one year by the number of women treated for complications due to spontaneous miscarriage or induced abortion care (2,625*12.2), multiplied by 100,000.

**Table 1:** **Classification of abortion complication severity level based on symptoms and clinical findings among women with abortion complications, Kenya 2012**

**Level of morbidity severity**	**Symptoms and signs**
Severe (requires at least one criteria)	Death
	Sepsis
	Temperature >37.9 degrees Celsius
	Evidence of mechanical injury/foreign body
	Shock
	Pulse >119 beats/minute
	Organ or system failure
	Generalized peritonitis
	Tetanus
Moderate (requires at least one criteria if subject isn’t in severe)	Offensive products of conception
	Temperature 37.3-37.9 degrees Celsius
	Localized peritonitis (tender uterus, discharge)
Low morbidity	Temperature <37.3 degrees Celsius
	No signs of infection
	No system or organ failure

*Note: A woman may have presented with several of these signs. For purposes of classification, complications in the most severe category determined the severity category a woman was assigned to. Adapted from: Rees et al.* [15]*.*

Descriptive and multivariable analyses were carried out using Stata (version 12.1) statistical software [16]. Descriptive results are presented as weighted percentages and unweighted number of cases for the various variables with Chi squared test values and level of significance. In the multivariable analyses, we assessed factors associated with level of complication severity. Variables assessed include age, education, marital status, place of residence, religion, gestational age, parity, history of previous abortion, history of interference with pregnancy, delay in seeking care, referral, pregnancy wantedness, occupation, level of health facility and geopolitical region.

Ordered logistic regression was used since the dependent variable (abortion complication severity level) has three ordinal categories (mild, moderate and severe complication). Proportional odds models which are often used for the analysis of ordinal dependent variables have a critical assumption of parallel slopes. This assumption implies that if a series of binary logistic regressions is conducted between the ordered categories, the coefficients (but not intercepts) would be the same across equations, save for sampling variability. However, this may not hold true for all variables being examined. The partial proportional odds model allows relaxation of the assumptions for those variables that violate the proportional slope assumption. This model can be fitted using a user written program *gologit2,* implemented in Stata statistical software [17]. The proportional slope assumption was assessed in preliminary analyses using *Brant* test and showed that overall; there was a violation of the parallel line assumption for several variables (X^2^ = 1,077.8, p-value < 0.001). Using gologit2 with the option *autofit,* the variables that do not conform to the parallel slope assumption were identified and additional estimates are given. Variables that conformed to the parallel slope assumption have one set of estimates as indicated. Further details on the methodology of partial proportional odds model and software for fitting the models can be found in several sources [17,18].

The study protocol was reviewed and approved by the ethical review boards of the Kenya Medical Research Institute on the 24^th^ of February 2012 (NON SSC No. 320) and the Institutional Review Board of the Guttmacher Institute.

## Results

Results in Table 2 show the proportions of women seeking post-abortion care with mild, moderate or severe post-abortion complications. Overall, 40.1% and 37.1% of women with abortions had moderate and severe post-abortion complications respectively. Divorced women, students, and farmers/unskilled women had significantly higher proportions of severe complications compared to the other categories (p < 0.05). Similarly, a higher proportion of women who reported that the index pregnancy was unwanted (at the time of conception), those who reported a history of interference with index pregnancy, and those whose gestational age was more than 12 weeks had higher proportions of severe complications compared to their counterparts (p < 0.05). Lastly, women who experienced longer delays in reaching a health care facility, those referred from other facilities and those managed at lower level facilities had disproportionately more severe post-abortion complications (p < 0.05).

**Table 2:** **Abortion complication severity distribution by socio-demographic and other characteristics**

	**Level of morbidity severity**				
**Variables**	**Low**	**Moderate**	**Severe**	**Number**	Uncorrected Chi2; design-based F*; p value
Age category					
10-19 years	16.7	38.2	45.1	334	23.05; 1.94; p = 0.1068
20-24 years	24.1	41.5	34.4	807	
25 + years	24.2	40.1	35.8	1,476	
Residence					
Urban	23.6	39.9	36.5	1,386	0.75; 0.05; p = 0.9371
Rural	22.3	40.3	37.4	1,235	
Marital status					
Never married	21.6	33.8	44.7	757	86.80; 7.02; p = 0.0001
Married	24.5	44.1	31.5	1,698	
Divorced	14.4	29.9	55.7	162	
Education					
No education	21.7	43.8	34.5	158	
Primary	19.3	40.5	40.2	961	
Secondary	24.0	38.3	37.6	948	36.40; 1.73; p = 0.1196
Post-secondary	30.7	41.0	28.2	543	
Occupation					
Farmer/unskilled	18.6	37.0	44.4	587	
Skilled/cleric	28.4	42.8	28.8	683	
Student	18.3	34.6	47.1	383	66.30; 3.28; p = 0.0051
Unemployed	24.2	42.5	33.4	963	
Religion					
Christians	23.3	39.1	37.6	2,367	
Muslims	22.5	46.0	31.5	216	21.9; 0.90; p = 0.4840
Others	10.1	53.3	36.7	42	
Parity					
1-2	22.9	42.0	35.1	1,375	
3-4	24.9	37.2	37.9	787	11.50; 0.87; p = 0.4722
5+	19.9	39.6	40.5	457	
Previous abortion					
Zero	22.9	40.3	36.8	2,027	0.14; 0.01; p = 0.9763
1+ previous abortions	22.8	39.7	37.5	587	
Pregnancy wanted-ness					
Wanted then	27.9	49.3	22.8	1,081	
Wanted later	21.2	37.4	41.4	645	207.49; 9.42; p < 0.0001
Did not want	18.8	29.8	51.4	800	
Don’t know	16.5	52.9	30.6	81	
Interference/induced					
No	27.1	46.7	26.2	1,886	303.26; 43.19; p < 0.0001
Yes	14.7	27.3	58.0	722	
Gestation age					
<=12 weeks	26.1	41.1	32.8	1,652	45.15; 6.88; p = 0.0011
>12 Weeks	18.1	38.6	43.3	973	
Delay					
<=6 hours	35.1	43.4	21.5	506	
7-48 hours	20.5	48.1	31.4	724	175.10; 8.62; p <0.0001
3-7 days	18.9	38.8	42.4	865	
More than 7 days	17.7	28.6	53.7	381	
Referred					
Not referred	23.7	42.6	33.6	1,823	45.10; 6.84; p = 0.0022
Referred	20.1	33.0	46.9	791	
Level of health facility					
Level 2	13.9	42.3	43.9	228	
Level 3	28.8	30.6	40.6	796	
Level 4	23.4	47.7	29.0	895	150.80; 4.59; p = 0.0006
Level 5	35.4	42.0	22.5	530	
Level 6	8.0	61.4	30.7	176	
Region					
Central & Nairobi	24.3	37.1	38.6	838	
Coast & North Eastern	19.1	57.3	23.6	399	
Eastern	37.8	30.5	31.7	362	106.46; 2.25; p = 0.0322
Nyanza & Western	19.6	38.7	41.7	505	
Rift valley	22.8	37.5	39.7	521	
Total	22.9	40.1	37.1	2625	

*Rao and Scott second-order corrected Pearson statistic.

Table 3 shows the uterine evacuation procedures carried out on the PAC clients that were captured in the survey by facility and clinician characteristics, geographical location and gestational age. Overall, most (65%) post-abortion clients were evacuated using manual or electric vacuum aspiration (MVA/EVA) with higher percentages in higher level health care facilities. About 8% of post-abortion cases were treated using misoprostol with a similar percentage treated with dilation and curettage. MVA/EVA were more common procedures in private-for-profit facilities (78%) followed by government owned facilities (64%). While dilation and curettage (D&C) was primarily performed by medical officers, MVA was mainly performed by clinical officers and obstetricians. A higher proportion (83%) of women with abortion complications in Central and Nairobi provinces were treated using MVA/EVA compared to 48% in Eastern province. Use of D&C was more prevalent in Eastern and Rift valley provinces while Nyanza and Western provinces had the highest proportion of women with abortion managed with misoprostol (11%). About 75% of early abortions (gestational age < = 12 weeks) were managed with MVA/EVA but a sizeable proportion of late abortions (51%) were also treated using the same procedure, which is not recommended in the current clinical practice of managing abortions [19].

**Table 3:** **Evacuation procedures distribution by health facility, health care provider and other characteristics**

	**D & C**	**MVA/EVA**	**Misoprostol**	Other*	**Number (unweighted)**
**Variables**	**(%)**	**(%)**	**(%)**	**(%)**	
Level of facility					
Level 2	4.0	43.1	14.4	38.5	119
Level 3	4.4	71.2	6.2	18.2	648
Level 4	14.6	69.8	5.5	10.1	768
Level 5	5.3	81.6	3.7	9.4	485
Level 6	3.6	94.0	1.2	1.2	166
Health facility ownership					
Public	5.9	64.2	4.3	25.6	1,434
Private-for-profit	8.7	78.4	6.6	6.2	397
Private-not-for-profit	14.3	51.2	21.3	13.2	355
Cadre of health worker					
Obstetrician/Gynecologist	31.8	60.4	4.9	2.9	107
Medical officer	32.6	53.6	7.3	6.5	402
Clinical officer	3.0	82.1	6.5	8.3	1,076
Nurse	2.2	54.6	5.2	37.9	559
Trained midwife	0.0	36.2	38.1	25.7	41
Region					
Central & Nairobi province	3.4	82.6	6.4	7.7	708
Coast & North Eastern province	2.8	64.2	6.7	26.3	330
Eastern province	18.7	48.3	1.5	31.6	278
Nyanza & Western province	3.1	61.7	11.3	23.8	437
Rift valley province	21.3	55.7	5.7	17.3	433
Gestational age					
<=12 week	6.0	74.7	3.4	15.9	1,419
>12 Week	11.0	50.8	14.1	24.1	767
Total	7.9	65.4	7.6	19.1	2,186

D&C- dilation and curettage; MVA- manual vacuum aspiration; EVA- electric vacuum aspiration. *The “Other” had use of forceps and finger/hand.

Table 4 shows the distribution of other forms of post-abortion client-management by severity level of complications. About 84% of post-abortion clients received analgesics, with a slightly higher proportion (87%) among those with severe complications (p < 0.05). About 91% of those with mild complication received antibiotics compared to 94% among those with severe complication. About 64% of abortion clients received oxytocics after evacuation procedure and on average a higher proportion of women with severe complications stayed in health care facilities longer than 12 hours (p < 0.05).

**Table 4:** **Post evacuation management and outcome by severity level**

**Variables**	**Low**	**Moderate**	**Severe**	**Total**	Uncorrected Chi2; design-based F*; p value
Received analgesics	*n = 585*	*n = 972*	*n = 630*	*N = 2187*	21.41; 2.53; p = 0.0886
Yes	83.9	79.7	87.2	83.5	
Received antibiotics	*n = 719*	*n = 1128*	*n = 768*	*N = 2615*	21.30; 3.99; p = 0.0202
Yes	90.9	93.2	96.1	93.7	
Received IV fluids	*n = 719*	*n = 1119*	*n = 767*	*N = 2605*	63.22; 7.84; p = 0.0005
Yes	38.9	37.3	52.4	43.3	
Received blood	*n = 715*	*n = 1120*	*n = 766*	*N2601*	43.83; 7.58; p = 0.0018
Yes	2.8	2.4	7.6	4.5	
Received oxytocics	*n = 713*	*n = 1119*	*n = 765*	*N = 2597*	5.25; 0.47; p = 0.6209
Yes	60.5	64.2	65.7	63.9	
Outcome	*n = 720*	*n = 1130*	*n = 770*	*N = 2620*	
Discharged	90.3	86.5	80.7	85.2	62.25; 2.10; p = 0.0418
Died	0.0	0.0	0.7	0.2	
Still Hospitalized	2.4	1.9	4.8	3.1	
Absconded	2.5	1.6	3.0	2.3	
Referred	4.8	10.1	10.9	9.2	
Stay in facility	*n = 719*	*n = 1126*	*n = 765*	*N = 2610*	
Less than 12 hours	65.5	68.0	59.7	64.3	55.46; 4.31; p = 0.0032
12-24 hour	25.9	25.5	24.9	25.4	
More than 24 hours	8.6	6.5	15.4	10.3	

*Rao and Scott second-order corrected Pearson statistic. Note “n” varies across variables.

Among those who sought care for abortion-related complications in the sampled health facilities, an estimated 85 women died in 2012. The health facility based case fatality rate was calculated as 266 deaths per 100,000 post-abortion care clients.

Table 5 shows results from regression models examining factors associated with level of severity of post- abortion complications. In the unadjusted models, ordered logistic regression models were fitted (panel I). Panel II and III have results from partial proportional odds regression models. Panel II has estimates of low complication severity contrasted against moderate and severe complication while panel III has estimates of mild and moderate severity contrasted against severe complication. Where the parallel slope assumption was not violated, estimates in Panel II and III are the same while for those where the assumption was violated, the corresponding estimates are indicated (panel III).

**Table 5:** **Factors associated with abortion complication severity; results from ordered logistic (I) and partial proportional odds regression models (II & III)**

**Variables**	**Unadjusted models**	**Adjusted models**	
	**OR (95% CI): (I)**	**OR (95% CI): low vs. moderate/severe (II)**	**OR (95% CI): low/moderate vs. severe (III)**
Age category (ref = 10-19 yrs)		Low	
20-24 yrs	0.64** (0.46-0.88)	0.89 (0.62-1.27)	*§*
25 + yrs	0.66* (0.48-0.92)	0.95 (0.63-1.44)	*§*
Residence (ref = Urban)			
Rural	1.05 (0.73-1.52)	0.82 (0.56-1.20)	*§*
Marital status (ref = Never married)			
Married	0.65** (0.50-0.86)	1.20 (0.82-1.76)	*§*
Divorced	1.62 (0.90-2.94)	1.32 (0.69-2.53)	*§*
Education (ref = No education)			
Primary	1.23 (0.77-1.95)	0.99 (0.56-1.74)	*§*
Secondary	1.03 (0.63-1.70)	0.74 (0.41-1.34)	*§*
Post-secondary	0.70 (0.42-1.15)	0.61 (0.30-1.23)	*§*
Occupation (ref = Farmer/unskilled)			
Skilled/cleric	0.53** (0.35-0.82)	0.81 (0.53-1.24)	*§*
Student	1.09 (0.73-1.64)	0.95 (0.60-1.49)	*§*
Unemployed	0.66* (0.45-0.96)	0.87 (0.62-1.23)	*§*
Religion (ref = Christians)			
Muslims	0.87 (0.0.57-1.32)	1.15 (0.69-1.91)	*§*
Others	1.27 (0.64-2.51)	1.09 (0.46-2.58)	*§*
Parity (ref = 1-2)			
3-4	1.03 (0.79-1.34)	0.84 (0.60-1.19)	1.18 (0.85-1.65)
5+	1.23 (0.91-1.67)	0.97 (0.63-1.49)	*§*
Previous abortion (ref = Nil)			
1+ previous abortions	1.02 (0.68-1.54)	0.88 (0.63-1.23)	*§*
Pregnancy timing (ref = Wanted then)			
Wanted later	1.87*** (1.37-2.56)	1.43* (1.01-2.03)	*§*
Did not want	2.66*** (1.94-3.65)	1.33 (0.87-2.04)	2.03*** (1.36-3.01)
Don’t know	1.55 (0.81-2.97)	1.35 (0.71-2.56)	*§*
Interference/induced (ref = No)			
Yes	3.32*** (2.45-4.49)	1.44 (0.88-2.37)	2.39*** (1.72-3.34)
Gestational age (ref = equal or less weeks)			
>12 Weeks	1.58*** (1.23-2.01)	1.65*** (1.24-2.21)	*§*
Delay (ref = <=6 hours)			
7-48 hours	1.81*** (1.28-2.55)	2.12*** (1.42-3.17)	1.34 (0.89-2.03)
3-7 days	2.52*** (1.69-3.78)	2.01*** (1.34-2.99)	*§*
More than 7 days	3.69*** (2.27-6.00)	2.35*** (1.45-3.79)	*§*
Referred (ref = Not referred)			
Referred	1.56** (1.12-2.17)	1.10 (0.81-1.48)	1.52* (1.10-2.10)
Level of health facility (ref = Level II)			
Level III	0.67 (0.40-1.11)	0.52* (0.30-0.88)	1.11 (0.70-1.78)
Level IV	0.56** (0.37-0.82)	0.70 (0.45-1.09)	*§*
Level V	0.36*** (0.24-0.54)	0.46** (0.29-0.73)	*§*
Level VI	0.79 (0.54-1.15)	2.54*** (1.53-4.21)	0.81 (0.31-2.07)
Region (ref = Central and Nairobi)			
Coast & North Eastern	0.77 (0.46-1.31)	0.78 (0.50-1.21)	*§*
Eastern	0.60 (0.30-1.20)	0.59 (0.31-1.13)	*§*
Nyanza & Western	1.21 (0.68-2.15)	0.95 (0.58-1.58)	*§*
Rift valley	1.06 (0.60-1.88)	1.17 (0.74-1.86)	*§*

*p < 0.05, **p < 0.01, ***p < 0.001; *§Parallel slope assumption not violated on these variables, estimates same as those in (II). OR = odds ratio, CI = confidence interval.*

Care-seeking, gestational age and wantedness of pregnancy were associated with severity of complications. Clients who reported for health care late had higher odds of being in a higher level of complication severity category compared to those who came early for management. A delay of more than 6 hours was associated with at least 2 times higher odds of having a severe complication compared to those who came within 6 hours from onset of symptoms. Delays of between 7 to 48 hours; 3–7 days and more than 7 days were associated with 2.12 (1.42-3.17), 2.01 (1.34-2.99) and 2.35 (1.45-3.39) times higher odds of having a moderate or severe complication respectively. Women whose pregnancy was more than 12 weeks gestation had a 65% higher odds (OR = 1.65, 95% CI 1.24-2.21) of having moderate or severe complication compared to those whose pregnancy was 12 weeks or less. Women whose pregnancy was mistimed or unwanted had higher odds of having moderate or severe complication compared to those whose pregnancy was wanted then. Compared to those who did not report interfering with the pregnancy, women who reported interfering with their pregnancy had 44% higher odds of having a moderate complication (non-significant), but the odds were higher and significant for getting severe complication (OR = 2.35, 95% CI 1.72-3.34).

With the exception of level VI facilities, women seen in higher level facilities (III to V) had lower odds of having a moderate or severe complication compared to those managed in level II facilities. Women attending level VI facilities had higher odds of having a moderate complication compared to those in level II. On the other hand, women who were referred to a facility for treatment from another facility were more likely to have a severe complication compared to those who had not been referred (OR = 1.52, 95% CI 1.10-2.10). Individual socio-demographic characteristics such as age, religion, place of residence, education and occupation were not associated with level of complication severity.

## Discussion

### Main findings

Over three quarters (77%) of Kenyan women seeking PAC had moderate to severe complications ranging from localized peritonitis to sepsis and death. This proportion was much higher than has been seen in other countries such as Malawi (28%) and Ethiopia (41%) [14,20]. Furthermore, the current estimate is substantially higher than that from 2002 where 44% of women presented to hospitals with moderate to severe complications [12,14,20]. The current estimate might be a reflection of worsening state of reproductive health access for Kenyan women, but may also be due to selection bias as the 2003 study was limited to higher level public health care facilities. Furthermore, this study found that 37% of women had severe complications, which was higher than the 28% estimated ten years ago [12]. Safe TOPs, when conducted by a trained provider in sanitary conditions, generally have very low rates of complications compared to those observed in this study [19]. The high proportion of women presenting with moderate to severe complications and high case fatality rate indicates that unsafe abortion continues to pose a major public health challenge in Kenya.

Severe complications were associated with delays in seeking health care, being managed in a lower level facility, and being referred to an upper level facility, and these are all interconnected and related factors. Health seeking delays are typically characterized into three stages- delays in recognizing a complication and deciding to seek care, traveling to a health facility, and receiving appropriate care upon arrival at a health facility [21-23]. Studies have shown that delays in receiving health care contribute to maternal morbidity and mortality [21,23]. Delays in seeking care occur due to a number of factors including lack of knowledge about complication or low perceived seriousness of complications, perceptions about services, access to transportation and ability to pay for treatment [23]. Even after arriving at a health care facility, there might be delays in getting the right treatment due to shortages in staffing, equipment, drugs, and poor attitude of health care providers [21,24,25]. These delays may result in worsening of an otherwise easily treatable complication, for example, through excessive blood loss, and or spread of infection to other parts of body resulting into severe complication and death. The effects of delays seen here are net of whether one was referred or not. The effect observed for referred cases does not mean that referral is responsible for the more severe forms of complication but rather that more complicated cases are more likely to be referred.

Severe complications were also associated with late gestational age and women reporting a history of interfering with the pregnancy. These findings are as expected and are in line with previous studies in Kenya over the past decade [12]. Recent studies of the sociocultural context of abortion in Kenya have documented a high prevalence of unintended pregnancies- with up to one in four parous women reporting their most recent pregnancy as unintended. Higher proportions of unwanted pregnancies are more common among single women, and those with higher parity in slum settlements [26]. To guard against potential consequences of unwanted or mistimed pregnancies, many women in this situation may opt to terminate their pregnancy.

### Strengths and weaknesses

The most recent study on post-abortion complications in Kenya was conducted in 2003 and was based on a sample of public health facilities. The current study used data from a nationally- representative sample of public and private health facilities. This study therefore provides a more complete picture of post-abortion care provision in Kenya. As opposed to review of medical records, this study was prospective in nature with every abortion case providing a full account of events as outlined in the questionnaires. This resulted in a near complete account of events for each woman seen. The data collection tools used in this study had been used elsewhere and had received extensive reviews and revision yielding data comparable to that of similar studies [12,14,15,27].

While our study had several strengths there were also weaknesses that might have impacted on the accuracy of the results. Data collection was facility-based and did not capture women who were in need of PAC and did not present to health facilities for care. Some of the women with very severe complication may have died without reaching a facility and as such our estimates of severity and case fatality rate might be on the lower side. In addition, some of the mild and moderate cases may not have come to seek care as these might have been managed at home and thus leaving higher severity level complications presenting at health care facilities. If this happened, the severe complication clients would be over represented. Also, while we assessed delay in reaching a health facility for care, this study did not assess delay between arrival and receiving care. This is an important aspect that would have given us a fuller understanding of quality of care in health care facilities. However, in spite of these limitations, this study provides an updated national picture of the state of unsafe abortion and its contribution to maternal morbidity in Kenya.

### Interpretation

Over three quarters of abortion complications were moderate or severe and this calls for urgent and correct treatment to avert life-long complication and death. While results indicate that MVA/EVA is the most common procedure currently used for the treatment of post-abortion complications, there are gaps. For example, MVA was seen to be used in late gestation abortions while D & C usage also remains prevalent. Misoprostol for post-abortion care has shown efficacy ranging between 95-99% and has been encouraged as an appropriate uterine evacuation method for all countries, and specifically in low resource settings [19,28]. Misoprostol for post-abortion care was only introduced to five public hospitals in Kenya between 2009 and 2010. The emerging evidence that misoprostol was used to treat 8% of all PAC cases nationally indicates its growing availability to facilities in Kenya following its registration in 2008 [28]. Although dilation and curettage is not recommended by the WHO, it is still widely used for uterine evacuation in the country, and in the Eastern and Rift valley provinces. This indicates critical inequities in provision of quality abortion care, highlighting the need to strengthen the implementation of the Standards and Guidelines on abortion care in Kenya [29].

While the Kenya Standards and Guidelines indicate that all PAC patients should receive analgesics, 16% of women in this study did not receive them [29]. In addition, 35% of patients with low severity of complications stayed longer than 12 hours in the facility. Given that low severity was defined as having no infection and no other form of complication, this means that many clients with mild complications might be staying longer than necessary in health care facilities. As such, it is likely that the limited resources are not being used efficiently in the public sector and clients in private facilities could be overpaying for the services.

Some of the factors associated with more severe forms of complication are amenable to intervention from the community and health provider perspectives. For example, delay in receiving care can be tackled through public education, provision of emergency transportation and improving provider efficiency in handling clients arriving at facilities for care. Also, through public education, the dangers of attempting to induce abortion, especially for pregnancies more than 12 weeks and by untrained personnel and in unsanitary premises, can be highlighted. The need to avoid unwanted pregnancies can also be emphasized through public education and improving demand and access to family planning commodities. The tendency to have more severe complications in level II compared to levels III to V might be related to quality of care in terms of trained staff and equipment in the higher level facilities. On the other hand, the higher risk of severe complication in level VI might be related to referrals from the lower level facilities of the very complicated cases from around the country.

Kenya continues to experience a high abortion complication fatality rate of 266 deaths per 100,000 unsafe abortions. According to WHO estimates, the unsafe abortion case fatality rate in developed regions is around 30 deaths per 100,000 unsafe abortions and 530 deaths per 100,000 unsafe abortions in Eastern Africa [1]. A recent study in Malawi estimated a case-fatality rate of 387 deaths per 100,000 unsafe abortions [30]. The high case-fatality rates indicate high contribution of abortion complication to maternal mortality and partly explain its persistently high levels in Kenya.

## Conclusion

Moderate and severe post-abortion complications are common among Kenyan women and many are not well managed. Most severe complications were from late gestation abortions, those that were unsafely induced, and those that delayed reaching a health care facility. Taken together, these findings call attention to the urgent need for concerted efforts to combat the impact of unsafe TOP on women in Kenya. We recommend that all facilities offering post-abortion care should implement the Standards and Guidelines to ensure that quality post-abortion care is available throughout Kenya. All PAC services need to include critical components of post-abortion family planning counseling and access to a range of family planning methods to further reduce unintended pregnancies. In addition, the special needs of young women who seek PAC need to be taken into account in programmes, as almost half of all women seeking PAC were aged 25 years or less. Urgent attention is also needed by policymakers and key stakeholders in women’s reproductive health to improve the impact of unsafe TOP on Kenyan women. By addressing the key factors identified here, such as delays in seeking care, late unsafe abortion, unsafe induction and inappropriate management of complications, we will go a long way in improving maternal health outcomes.
